# Estimates of the reproduction number for seasonal, pandemic, and zoonotic influenza: a systematic review of the literature

**DOI:** 10.1186/1471-2334-14-480

**Published:** 2014-09-04

**Authors:** Matthew Biggerstaff, Simon Cauchemez, Carrie Reed, Manoj Gambhir, Lyn Finelli

**Affiliations:** Epidemiology and Prevention Branch, Influenza Division, National Center for Immunization and Respiratory Diseases, Centers for Disease Control and Prevention, 1600 Clifton Road NE, MS A-32, Atlanta, 30333 Georgia; Mathematical Modelling of Infectious Diseases Unit, Institut Pasteur, Paris, France; National Center for Immunization and Respiratory Diseases, CDC, Atlanta, Georgia

**Keywords:** Reproductive number, Influenza, Pandemics, Zoonotic influenza

## Abstract

**Background:**

The potential impact of an influenza pandemic can be assessed by calculating a set of transmissibility parameters, the most important being the reproduction number (R), which is defined as the average number of secondary cases generated per typical infectious case.

**Methods:**

We conducted a systematic review to summarize published estimates of R for pandemic or seasonal influenza and for novel influenza viruses (e.g. H5N1). We retained and summarized papers that estimated R for pandemic or seasonal influenza or for human infections with novel influenza viruses.

**Results:**

The search yielded 567 papers. Ninety-one papers were retained, and an additional twenty papers were identified from the references of the retained papers. Twenty-four studies reported 51 R values for the 1918 pandemic. The median R value for 1918 was 1.80 (interquartile range [IQR]: 1.47–2.27). Six studies reported seven 1957 pandemic R values. The median R value for 1957 was 1.65 (IQR: 1.53–1.70). Four studies reported seven 1968 pandemic R values. The median R value for 1968 was 1.80 (IQR: 1.56–1.85). Fifty-seven studies reported 78 2009 pandemic R values. The median R value for 2009 was 1.46 (IQR: 1.30–1.70) and was similar across the two waves of illness: 1.46 for the first wave and 1.48 for the second wave. Twenty-four studies reported 47 seasonal epidemic R values. The median R value for seasonal influenza was 1.28 (IQR: 1.19–1.37). Four studies reported six novel influenza R values. Four out of six R values were <1.

**Conclusions:**

These R values represent the difference between epidemics that are controllable and cause moderate illness and those causing a significant number of illnesses and requiring intensive mitigation strategies to control. Continued monitoring of R during seasonal and novel influenza outbreaks is needed to document its variation before the next pandemic.

**Electronic supplementary material:**

The online version of this article (doi:10.1186/1471-2334-14-480) contains supplementary material, which is available to authorized users.

## Background

Annual influenza epidemics occur worldwide and cause substantial morbidity and mortality [1]. In the United States between 5% and 20% of the population are infected with influenza every year [2], resulting in between 3,000 and 49,000 influenza-associated deaths [3]. Influenza viruses are constantly changing either through the collection of minor point mutations or through major antigenic shifts. These major shifts can result in the introduction of novel influenza viruses into the human population to which humans have little or no immunity, causing pandemics [1]. Four influenza pandemics have occurred since the beginning of the 20^th^ century and have ranged widely in transmissibility and clinical severity [1, 4].

Recognizing that the characteristics of future pandemics will be difficult to predict given the mutability of the influenza virus and the range of morbidity and mortality experienced in previous pandemics, an approach to the early assessment of influenza pandemics has been developed relying on standardized measures of transmissibility and clinical severity [5]. An important transmissibility parameter identified is the reproduction number (R), which is defined as the average number of secondary cases generated per typical infectious case [6, 7]. R describes on average how many persons a case will infect, and a value of R greater than 1 indicates that the infection may grow or persist in the population while a value of R less than 1 indicates that the infection will decline in the population, although exceptions exist [7, 8]. Many methods to calculate R have been described that allow for the use of epidemiologic data from different epidemic time points [7]. Some examples include estimating R using the growth rate of the epidemic, the epidemic curve’s size and shape, the final attack rate, or by direct observation of disease transmission from one generation to the next [7]. The population susceptibility to the infection also affects the interpretation of R. If R is calculated in a population entirely susceptible to infection (or where an assumption about population susceptibility to infection is made), then R is known as the basic reproduction number (R_0_). In contrast, the effective reproduction number (R_E_) is calculated in a population with underlying immunity and accounts for a population’s reduced susceptibility to infection [9].

The value of R characterizes the final number infected in the absence of an intervention in homogeneously mixed populations, the herd immunity threshold, and, when coupled with the generation time, defined as the interval between infections in two consecutive generations, or the serial interval, defined as the interval between the onset of symptoms in two consecutive generations), the speed with which the disease spreads in the population [10–12]. Therefore, the magnitude of R plays an important role in the selection and aggressiveness of countermeasures (e.g. social distancing, treating ill individuals, or vaccination) required to slow transmission of the disease [10, 13].

Because R is used as a measure of transmissibility and informs the selection of different mitigation strategies, it is important to understand the range and uncertainty of published R values. In this paper, we investigate whether published estimates of R differ between pandemic, seasonal, and novel influenza, we compare values of R calculated in differing geographic regions and settings, and we explore the assumptions and limitations of the estimation methods of R.

## Methods

We performed a literature search using the PubMed database from 1950 to January 16, 2013. The following key terms were searched: “reproduction number and influenza”, “reproductive number and influenza”, “R_0_ and influenza”, “reproduction rate and influenza”, and “reproductive rate and influenza”. We limited our search to articles in English. We retained articles that estimated R for pandemic or seasonal influenza or for human infections with non-human influenza viruses (e.g. H5N1). For all studies retained, we abstracted the date of publication, the year, the geographic location where the data were collected, the influenza subtype, the study population, whether it was a confined setting, the wave of the observation (if during a pandemic), the estimated value of R, the method to identify influenza cases, and whether it was a R_0_ or R_E_. If multiple R values were provided, we provide the median and range. Since methods to estimate the reproduction number often require a value for the generation time or the serial interval, we also report those values [14]. We classified the method used to determine influenza-associated cases into two categories: laboratory confirmed, which required the use of confirmatory testing of respiratory or blood specimens, or unconfirmed, which relied on syndromic case definitions to identify cases and required no laboratory confirmation of illnesses.

Median R values and interquartile ranges (IQR) were reported for each pandemic and for the group of inter-pandemic seasonal epidemics. If a range of values was given for an individual study instead of a point estimate, the middle value of the range was used in the pandemic or epidemic median calculations.

## Results

**Figure 1:**
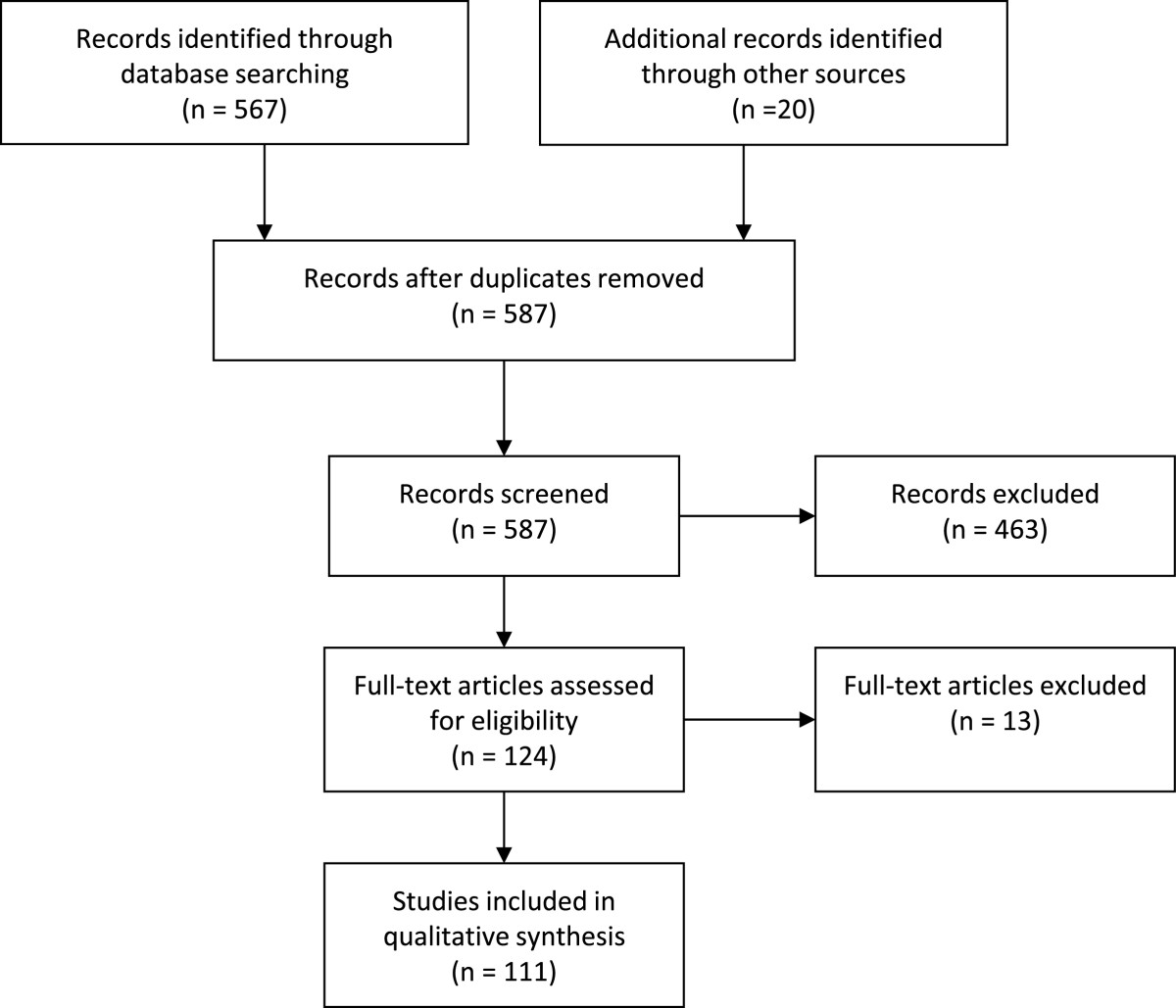
**PRISMA flowchart of the article selection for the reproductive number and influenza literature review.**

The search strategy initially identified 567 papers (Figure 1). Ninety-one papers were retained that estimated R for pandemic or seasonal influenza or for human infections with non-human influenza viruses (e.g. H5N1). Twenty additional papers were contributed by the references of the papers identified through the original search. In all, 111 articles were retained that presented original estimates of the reproduction number (summarized in Tables 1, 2, 3, 4, 5 and 6). Data provided in the tables are also available as .csv files in Additional files 1, 2, 3, 4, 5 and 6.

**Table 1:** **Reproduction numbers from the 1918 Influenza A/H1N1 Pandemic**

Location	Wave^a^	Study population	Mean GT/SI^b^	Reproduction Number (R)	95% CI^c^	Basic or effective	Case definition	Reference	Year published
Australia	1st	Community	2.6	1.80	1.6–2.0	Basic	Unconfirmed hospitalizations/deaths	[15]	2008
Brazil	1st	Community	4	2.68		Basic	Unconfirmed illness	[16]	2007
Canada	1st	Community	3	1.50	1.5–1.5	Basic	Unconfirmed deaths	[17]	2011
Canada	1st	Community	6	2.1	2.1–2.1	Basic	Unconfirmed deaths	[17]	2011
Colombia	1st	Community	3	1.4–1.5		Effective	Unconfirmed deaths	[18]	2012
Colombia	1st	Community	4	1.5–1.7		Effective	Unconfirmed deaths	[18]	2012
Denmark	1st	Community	2.6	2.2–2.4		Effective	Unconfirmed illness	[19]	2008
Denmark	1st	Community	4	2.8–3.0		Effective	Unconfirmed illness	[19]	2008
Denmark	1st	Community	2.6	2.8–4.0		Effective	Unconfirmed hospitalizations	[19]	2008
Denmark	1st	Community	4	3.6–5.4		Effective	Unconfirmed hospitalizations	[19]	2008
Italy	1st	Community	3	1.03	1.00–1.08	Basic	Unconfirmed hospitalizations	[20]	2011
Mexico	1st	Community	3	1.30		Effective	Unconfirmed deaths	[21]	2010
Peru	1st	Community	3	1.38	1.37–1.40	Effective	Unconfirmed deaths	[22]	2011
Switzerland	1st	Community	3.11	1.49	1.45–1.53	Basic	Unconfirmed hospitalizations	[23]	2006
Switzerland	1st	Community	3.4	1.50		Basic	Unconfirmed deaths	[24]	2009
United Kingdom	1st	Community	2.6	1.7		Basic	Unconfirmed deaths	[10]	2006
United Kingdom	1st	Community	4.1	2.10		Effective	Unconfirmed illness	[25]	2006
United Kingdom	1st	Community	6	2.00		Basic	Unconfirmed illness	[26]	2005
United Kingdom	1st	Community	NR	1.16–2.94		Effective	Unconfirmed illness	[27]	2010
United Kingdom	1st	Students	NR	1.43–5.36		Effective	Unconfirmed illness	[27]	2010
USA	1st	Community	4	1.34–3.21		Effective	Unconfirmed illness	[28]	2008
Various	1st	Community	4	1.2–3.0		Effective	Unconfirmed illness	[29]	2007
Various	1st	Community	4	2.1–7.5		Effective	Unconfirmed illness	[29]	2007
	1st	Sailors	4	4.97		Effective	Unconfirmed illness	[28]	2008
Canada	2nd	Community	3.6	2.26	1.95–2.63	Basic	Unconfirmed illness	[30]	2010
Canada	2nd	Community	3.6	1.49	1.42–1.55	Basic	Unconfirmed illness	[30]	2010
Canada	2nd	Community	3	2.40	2.4–2.5	Basic	Unconfirmed deaths	[17]	2011
Canada	2nd	Community	6	4.3	4.2–4.4	Basic	Unconfirmed deaths	[17]	2011
Denmark	2nd	Community	2.6	1.22–1.24		Effective	Unconfirmed illness	[19]	2008
Denmark	2nd	Community	4	1.29–1.33		Effective	Unconfirmed illness	[19]	2008
Denmark	2nd	Community	2.6	1.2–1.3		Effective	Unconfirmed hospitalizations	[19]	2008
Denmark	2nd	Community	4	1.3–1.4		Effective	Unconfirmed hospitalizations	[19]	2008
Germany	2nd	Community	1	1.58	0.03–10.3	Basic	Unconfirmed deaths	[31]	2007
Germany	2nd	Community	3	2.52	0.75–5.85	Basic	Unconfirmed deaths	[31]	2007
Germany	2nd	Community	5	3.41	1.91–5.57	Basic	Unconfirmed deaths	[31]	2007
Italy	2nd	Community	3	1.38	1.3–1.5	Basic	Unconfirmed hospitalizations	[20]	2011
Mexico	2nd	Community	3	1.30		Effective	Unconfirmed deaths	[21]	2010
New Zealand	2nd	Military	>1.5	1.3–3.1		Basic	Unconfirmed hospitalizations	[32]	2006
Switzerland	2nd	Community	2.28	3.75	3.6–3.9	Effective	Unconfirmed hospitalizations	[23]	2006
Switzerland	2nd	Community	3.4	2.40		Basic	Unconfirmed deaths	[24]	2009
United Kingdom	2nd	Community	3	1.39	1.36–1.43	Effective	Unconfirmed deaths	[33]	2008
United Kingdom	2nd	Community	6	1.84	1.75–1.92	Effective	Unconfirmed deaths	[33]	2008
United Kingdom	2nd	Community	6	1.55		Basic	Unconfirmed illness	[26]	2005
United Kingdom	2nd	Community	2.6	1.50		Basic	Unconfirmed deaths	[10]	2006
USA	2nd	Community	2.5	2.14		Basic	Unconfirmed deaths	[34]	2009
USA	2nd	Community	NR	2.20	1.55–2.84	Effective	Unconfirmed illness	[35]	2007
USA	2nd	Community	4	2.00	1.7–2.3	Effective	Unconfirmed deaths	[36]	2004
USA	2nd	Community	2.85	1.73		Effective	Unconfirmed deaths	[14]	2007
United Kingdom	3rd	Community	3	1.39	1.29–1.49	Effective	Unconfirmed deaths	[33]	2008
United Kingdom	3rd	Community	6	1.82	1.61–2.05	Effective	Unconfirmed deaths	[33]	2008
United Kingdom	3rd	Community	6	1.70		Basic	Unconfirmed illness	[26]	2005
Median reproduction number for the 1918 pandemic: 1.80; Interquartile range 1.47–2.27									

^a^The first wave of illnesses began in the Northern Hemisphere in the spring 1918 [1]. A second wave of more intense transmission occurred concurrently in North America, Europe, and Africa in the Fall of 1918 while a third and final wave of activity occurred in some areas of the world during the winter of 1919 [37].

^b^The generation time (GT) or serial interval (SI) assumed in the reproduction number estimation.

^c^Confidence interval.

NR = Not reported.

This table is also available as a .csv file as Additional file 1.

**Table 2:** **Reproduction numbers from the 1957 influenza A/H2N2 pandemic**

Location	Wave^a^	Study population	Mean GT/SI^b^	Reproduction number (R)	95% CI^c^	Basic or effective	Case definition	Reference	Year published
Netherlands	2nd	Community	3	1.39		Basic	Unconfirmed deaths	[38]	2010
United Kingdom	2nd	Community	2.6	1.70		Basic	Unconfirmed deaths	[10]	2006
United Kingdom	2nd	Community	3	1.5–1.6		Basic	Unconfirmed illness	[39]	2008
United Kingdom	2nd	Community	4	1.7–1.8		Basic	Unconfirmed illness	[39]	2008
United Kingdom	2nd	Community	4.1	1.50		Effective	Unconfirmed illness	[25]	2006
United Kingdom	2nd	Community	NR	1.65		Basic	Serology confirmed infection	[26]	2005
USA	2nd	Community	4	1.70		Basic	Unconfirmed illness	[40]	2004
Median reproduction number for the 1957 pandemic: 1.65; Interquartile range 1.53–1.70									

^a^The 1957 influenza A/H2N2 pandemic began in February 1957 in southern China and spread to Singapore and Hong Kong in April [1]. The virus was first isolated in the United States in June 1957 and was associated with a mild first wave of illnesses [1, 41]. The peak of the pandemic occurred during the second wave in the Northern Hemisphere in October 1957 and was followed by a third wave in January 1958.

^b^The generation time (GT) or serial interval (SI) assumed in the reproduction number estimation.

^c^Confidence interval.

NR = Not reported.

This table is also available as a .csv file as Additional file 2.

**Table 3:** **Reproduction numbers from the 1968 influenza A/H3N2 pandemic**

Location	Wave^a^	Study population	Mean GT/SI^b^	Reproduction number (R)	95% CI^c^	Basic or effective	Case definition	Reference	Year published
Hong Kong	1st	Community	2.95	1.89		Basic	Unconfirmed illness	[42]	1986
various	1st	Community	4	1.06–2.06		Basic	Serology; laboratory confirmed illness; unconfirmed illness	[43]	2010
various	1st	Confined settings	4	1.08–1.62		Basic	Serology; laboratory confirmed illness; unconfirmed illness	[43]	2010
United Kingdom	1st	Community	4.1	1.80		Effective	Unconfirmed illness	[25]	2006
United Kingdom	2nd	Community	NR	1.85		Effective	Serology confirmed infection	[26]	2005
various	2nd	Community	4	1.08–2.02		Effective	Serology; laboratory confirmed illness; unconfirmed illness	[43]	2010
various	2nd	Confined settings	4	1.43	1.23–1.63	Effective	Serology; laboratory confirmed illness; unconfirmed illness	[43]	2010
Median reproduction number for the 1968 pandemic: 1.80; Interquartile range 1.56–1.85.									

^a^The 1968 influenza A/H3N2 pandemic began in Hong Kong in July 1968. Large single waves of illness were reported in the Northern Hemisphere between September 1968 and April 1969 (with peaks occurring in December 1968–January 1969). Large single waves of illnesses were reported in the Southern Hemisphere between June and September 1969. Some countries in the Northern Hemisphere, such as the United Kingdom, did not have an outbreak of H3N2 until the winter of 1969–70.

^b^The generation time (GT) or serial interval (SI) assumed in the reproduction number estimation.

^c^Confidence interval.

NR = Not reported.

This table is also available as a .csv file as Additional file 3.

**Table 4:** **Reproduction numbers from the 2009 influenza A/H1N1 pandemic**

Location	Wave^a^	Study population	Mean GT/SI^b^	Reproduction number (R)	95% CI^c^	Basic or effective	Case definition	Reference	Year published
Mexico	0	Community	1.91	1.25	0.76–1.74	Basic	Laboratory confirmed illness	[44]	2011
Australia	1st	Community	2.8	1.50	1.50–2.70	Effective	Laboratory confirmed illness	[45]	2010
Australia	1st	Community	2.8	1.20	1.0–1.4	Effective	Laboratory confirmed illness	[45]	2010
Australia	1st	Community	2.9	2.40	2.3–2.4	Effective	Laboratory confirmed illness	[46]	2009
Australia, rural	1st	Community	2.9	1.28	1.26–1.30	Effective	Laboratory confirmed illness	[47]	2011
Australia, urban	1st	Community	2.9	1.26	1.22–1.30	Effective	Laboratory confirmed illness	[47]	2011
Canada	1st	Community	1.91	1.30	1.12–1.47	Basic	Laboratory confirmed illness	[48]	2010
Canada	1st	Community	2.78	2.21	1.98–2.50	Basic	Laboratory confirmed illness	[49]	2012
Canada	1st	Community	3.6	1.63	1.31–1.96	Basic	Laboratory confirmed illness	[48]	2010
Canada	1st	Community	4.31	1.31	1.25–1.38	Basic	Laboratory confirmed illness	[50]	2010
Chile	1st	Community	2.5	1.80	1.6–2.0	Effective	Unconfirmed emergency room visits	[51]	2010
Chile, central	1st	Community	3	1.32	1.27–1.37	Effective	Unconfirmed hospitalizations	[52]	2012
Chile, northern	1st	Community	3	1.19	1.13–1.24	Effective	Unconfirmed hospitalizations	[52]	2012
Chile, southern	1st	Community	3	1.58	1.45–1.72	Effective	Unconfirmed hospitalizations	[52]	2012
China	1st	Community	2.6	1.25	1.22–1.28	Effective	Laboratory confirmed illness	[53]	2012
China	1st	Community	4.31	1.53	1.45–1.60	Basic	Laboratory confirmed illness	[54]	2012
China	1st	Community	NR	1.68		Basic	Laboratory confirmed illness	[55]	2011
Hong Kong	1st	Community	3	1.70	1.6–1.8	Effective	Laboratory confirmed illness	[56]	2010
Hong Kong	1st	Community	3.2	1.45	1.4–1.5	Effective	Laboratory confirmed illness	[57]	2010
Israel	1st	Community	2.92	1.06	0.97–1.16	Effective	Laboratory confirmed illness	[58]	2011
Italy	1st	Community	2.6	1.30	1.23–1.32	Effective	Unconfirmed illness	[59]	2012
Japan	1st	School	1.9	2.30	2.0–2.6	Effective	Laboratory confirmed illness	[60]	2009
Japan	1st	Community	2.7	1.28	1.23–1.33	Effective	Laboratory confirmed illness	[60]	2009
Mexico	1st	Community	1.91	1.58	1.34–2.04	Basic	Unconfirmed illness	[61]	2009
Mexico	1st	Community	1.96	1.42		Basic	Unconfirmed illness	[62]	2010
Mexico	1st	Community	2.6	1.40	1.2–1.9	Basic	Laboratory confirmed illness	[61]	2009
Mexico	1st	Community	2.6	1.22	1.05–1.60	Basic	Laboratory confirmed illness	[61]	2009
Mexico	1st	Community	3	1.80	1.78–1.81	Effective	Unconfirmed illness	[63]	2011
Mexico	1st	Community	3	1.43	1.29–1.57	Effective	Laboratory confirmed illness	[64]	2009
Mexico	1st	Community	3.1	2.20	2.1–2.4	Effective	Laboratory confirmed illness	[65]	2009
Mexico	1st	Community	3.5	2.30	2.1–2.5	Basic	Laboratory confirmed illness	[11]	2009
Mexico	1st	Community	3.6	1.75	1.6–1.9	Basic	Seeding from Mexico	[66]	2009
Mexico	1st	Community	4.1	3.10	2.9–3.5	Effective	Laboratory confirmed illness	[65]	2009
Mexico City	1st	Community	3	1.72		Basic	Laboratory confirmed illness	[67]	2009
Morocco	1st	Community	2.3	1.44	1.32–1.56	Basic	Laboratory confirmed illness	[68]	2012
Morocco	1st	Community	2.7	1.40	1.34–1.48	Basic	Laboratory confirmed illness	[68]	2012
Netherlands	1st	Community	3	0.50		Effective	Laboratory confirmed illness	[69]	2009
New Zealand	1st	Community	2.7	1.25	1.07–1.47	Effective	Laboratory confirmed illness	[70]	2011
New Zealand	1st	Community	2.8	1.96	1.80–2.15	Effective	Laboratory confirmed illness	[71]	2009
New Zealand	1st	Community	2.8	1.55	1.16–1.86	Effective	Laboratory confirmed illness; unconfirmed illness	[72]	2010
North America	1st	Community	2.7	1.3–2.1		Basic	Laboratory confirmed illness	[73]	2010
Peru	1st	Community	2.8	1.37	1.33–1.41	Effective	Laboratory confirmed illness	[74]	2009
Peru	1st	Community	3	1.30	1.3–1.3	Effective	Unconfirmed illness	[75]	2011
Peru, Lima	1st	Community	3	1.70	1.6–1.7	Effective	Unconfirmed illness	[75]	2011
Singapore	1^st^	Dance club	1.91	1.9–2.1		Basic	Laboratory confirmed illness	[76]	2010
Singapore	1st	Military	NR	1.91	1.50–2.36	Effective	Laboratory confirmed and unconfirmed illness	[77]	2010
South Africa	1st	Community	2.3	1.43	1.38–1.49	Effective	Laboratory confirmed illness	[78]	2012
South Africa	1st	Community	2.78	1.47	1.30–1.72	Effective	Laboratory confirmed illness	[78]	2012
South Africa	1st	Community	2.78	1.42	1.20–1.71	Effective	Laboratory confirmed illness	[78]	2012
Southern Hemisphere	1st	Community	1.9	1.16–1.53		Effective	Laboratory confirmed illness	[79]	2010
Southern Hemisphere	1st	Community	2.60	1.33	1.28–1.45	Basic	Laboratory confirmed and unconfirmed illness	[80]	2011
Taiwan	1st	Community	1.91	1.14	1.04–1.25	Effective	Laboratory confirmed illness	[81]	2011
Taiwan	1st	Community	NR	1.16	0.98–1.34	Effective	Serology confirmed infection	[82]	2011
Thailand	1st	Community	1.9	1.78	1.67–1.89	Basic	Laboratory confirmed illness	[83]	2009
Thailand	1st	Community	2.6	2.07	1.92–2.22	Basic	Laboratory confirmed illness	[83]	2009
United Kingdom	1st	School	2.2	1.33	1.11–1.56	Effective	Laboratory confirmed illness	[84]	2012
United Kingdom	1st	Community	2.5	1.44	1.27–1.63	Effective	Laboratory confirmed illness	[85]	2009
USA	1st	Community	2.2	1.70	1.4–2.1	Basic	Laboratory confirmed illness	[86]	2009
USA	1st	Community	2.6	2.20	1.4–2.5	Basic	Laboratory confirmed illness	[86]	2009
USA	1st	School	2.7	3.30	3.0–3.6	Effective	Unconfirmed illness	[87]	2009
USA	1st	Community	3.5	1.3–2.0	1.0–2.2	Basic	Laboratory confirmed illness	[11]	2009
USA	1st	Camp attendees	7	2.20	1.4–3.3	Effective	Unconfirmed illness	[88]	2011
Vietnam	1st	Community	1.9	1.50	1.5–1.6	Basic	Laboratory confirmed illness	[89]	2010
Vietnam	1st	Community	3.6	2.00	1.9–2.2	Basic	Laboratory confirmed illness	[89]	2010
worldwide	1st	Community	2.67	1–2		Effective	Laboratory confirmed illness	[90]	2011
China	2nd	Community	4	1.66	1.27–2.05	Effective	confirmed hospitalizations	[91]	2012
China	2nd	Community	4.3	1.70	1.4–1.9	Effective	Laboratory confirmed illness	[92]	2010
France	2nd	Military	2.9	1.5–1.6		Effective	Unconfirmed illness	[93]	2012
Iran	2nd	school	NR	1.28	1.05–1.54	Basic	Unconfirmed illness	[94]	2012
Italy	2nd	Community	2.5	1.33		Effective	Unconfirmed illness	[95]	2011
Japan	2nd	Community	3	1.48	1.41–1.56	Effective	Unconfirmed illness	[96]	2012
Mexico	2nd	Community	3	1.62	1.61–1.63	Effective	Unconfirmed illness	[63]	2011
Reunion Island	2nd	Community	2.8	1.26	1.08–1.49	Effective	Unconfirmed illness	[97]	2010
Taiwan	2nd	Community	1.91	1.02	1.01–1.02	Effective	Laboratory confirmed illness	[81]	2011
Taiwan	2nd	Community	NR	1.87	1.68–2.06	Effective	Serology confirmed infection	[82]	2011
United Kingdom	2nd	Community	2.5	1.30	1.2–1.5	Effective	Laboratory confirmed illness	[98]	2010
Mexico	3rd	Community	3	1.24	1.23–1.24	Effective	Unconfirmed illness	[63]	2011
various		Community	NR	1.30	1.1–1.4	Effective	Serology confirmed infection	[99]	2012
Median reproduction number for the 2009 pandemic: 1.46; Interquartile range 1.30–1.70									

^a^The 2009 influenza A/H1N1 pandemic began in Mexico in the late winter or early spring of 2009 [44]. The United States and the United Kingdom experienced a first wave of illnesses in the Spring of 2009 followed by a second wave during the Fall of 2009 [4]. However, unlike these three countries, a number of countries, especially in the Southern Hemisphere, only experienced a single wave of illnesses [100].

^b^The generation time (GT) or serial interval (SI) assumed in the reproduction number estimation.

^c^Confidence interval.

NR = Not reported.

This table is also available as a .csv file as Additional file 4.

**Table 5:** **Reproduction numbers from seasonal influenza epidemics**

Year(s)	Type/Subtype	Study population	Mean GT/SI^a^	Reproduction number (R)	95% CI^b^	Basic or effective	Case definition	Reference	Year published
1889–1890	H3N8?	USA & Europe	2.6	2.10	1.9–2.4	Basic	Unconfirmed deaths	[101]	2010
1948–1949	H1N1	Canada	4.1	1.30		Effective	Unconfirmed illness	[25]	2006
1949–1950	H1N1	Canada	4.1	1.50		Effective	Unconfirmed illness	[25]	2006
1950–1951	H1N1	Canada & UK	4.1	2.00	1.9–2.5	Effective	Unconfirmed deaths	[25]	2006
1958–1973	H2N2; H3N2; B	United Kingdom	4.48	3.9–7.1		Effective	Unconfirmed illness	[102]	1979
1972–2002	H1N1/H3N2/B	Australia	5.5	1.30		Effective	Unconfirmed deaths	[103]	2008
1972–2002	H1N1/H3N2/B	France	5.5	1.30		Effective	Unconfirmed deaths	[103]	2008
1972–2002	H1N1/H3N2/B	USA	5.5	1.30		Effective	Unconfirmed deaths	[103]	2008
1972–2002	H1N1/H3N2/B	USA; France; Australia	5.5	1.30	1.2–1.4	Effective	Unconfirmed deaths	[103]	2008
1975–2004	H1N1/H3N2/B	Norway	6	1.06–1.69		Effective	Unconfirmed deaths	[104]	2010
1976–1981	H1N1/H3N2/B	USA	2.6	1.70		Basic	Serology confirmed infection	[10]	2006
1976–1981	H1N1/H3N2/B	USA	4.1	1.16		Basic	Serology confirmed infection	[105]	2000
1977–1978	H1N1	United Kingdom	2.2	4.38		Basic	Unconfirmed illness	[106]	2005
1977–1978	H1N1	United Kingdom	3	21.00		Basic	Unconfirmed illness	[13]	2004
1977–1978	H1N1	United Kingdom	4.70	16.90		Basic	Unconfirmed illness	[106]	2005
1984–1985	H1N1/H3N2	France	2.49	1.37		Effective	Unconfirmed illness	[107]	1988
1985–2005	H1N1/H3N2/B	United Kingdom	2.2	1.6–2.1		Basic	Unconfirmed illness	[108]	2010
1985–2005	H1N1/H3N2/B	United Kingdom	2.7	1.6–2.5		Basic	Unconfirmed illness	[109]	2012
1985–2006	H1N1/H3N2/B	France	2.4	1.4–1.7	1.3–1.8	Basic	Unconfirmed illness	[110]	2008
1996–2006	H1N1/H3N2/B	Brazil	3	1.03	1.02–1.04	Effective	Unconfirmed deaths	[111]	2010
1998–1999	H3N2	Israel	3	1.14		Effective	Unconfirmed illness	[112]	2011
1998–1999	H3N2	Israel	3	1.16		Effective	Unconfirmed illness	[112]	2011
1998–1999	H3N2	USA	3	1.18	1.05–1.25	Effective	Laboratory confirmed illness	[113]	2009
1998–2009	H1N1/H3N2/B	Israel	2.5	1.17–1.62		Effective	Unconfirmed illness	[114]	2012
1999–2000	H3N2	Israel	3	1.16		Effective	Unconfirmed illness	[112]	2011
1999–2000	H3N2	Israel	3	1.18		Effective	Unconfirmed illness	[112]	2011
1999–2006	Seasonal H1N1	Taiwan	2	1.19	0.76–1.86	Basic	Confirmed and unconfirmed illness	[115]	2010
1999–2006	H3N2	Taiwan	3	1.41	0.92–2.19	Basic	Confirmed and unconfirmed illness	[115]	2010
1999–2006	B	Taiwan	3	1.07	0.69–1.69	Basic	Confirmed and unconfirmed illness	[115]	2010
2000–2001	H1N1	Israel	3	1.12		Effective	Unconfirmed illness	[112]	2011
2000–2009	H1N1/H3N2/B	Italy	4	1.17–1.36		Effective	Unconfirmed illness	[116]	2012
2001–2002	H3N2	Israel	3	1.25		Effective	Unconfirmed illness	[112]	2011
2001–2002	H3N2	Israel	3	1.27		Effective	Unconfirmed illness	[112]	2011
2003–2004	H3N2	Israel	3	1.19		Effective	Unconfirmed illness	[112]	2011
2003–2004	H3N2	Israel	3	1.21		Effective	Unconfirmed illness	[112]	2011
2003–2004	H3N2	Switzerland	2.6	1.2–1.3		Effective	Unconfirmed illness	[117]	2011
2004–2005	H3N2	Israel	3	1.25		Effective	Unconfirmed illness	[112]	2011
2004–2005	H3N2	Israel	3	1.25		Effective	Unconfirmed illness	[112]	2011
2004–2005	unspecified	Taiwan	4.1	1.00		Effective	Unconfirmed deaths	[118]	2010
2004–2005	H3N2	USA	7	1.20	1.1–1.3	Effective	Laboratory confirmed illness	[119]	2008
2006–2007	H3N2	Israel	3	1.28		Effective	Unconfirmed illness	[112]	2011
2006–2007	H3N2	Israel	3	1.33		Effective	Unconfirmed illness	[112]	2011
2007–2008	H3N2	Israel	3	1.25		Effective	Unconfirmed illness	[112]	2011
2007–2008	H3N2	Israel	3	1.29		Effective	Unconfirmed illness	[112]	2011
2011/12	H1N1	Mexico	3	1.20		Effective	Laboratory confirmed hospitalizations	[120]	2012
2011/12	H1N1	Mexico	3	1.20		Effective	Laboratory confirmed hospitalizations	[121]	2012
2011/12	H1N1	Mexico	4	1.30		Effective	Laboratory confirmed hospitalizations	[121]	2012
Median reproduction number for seasonal influenza: 1.28; Interquartile range 1.19–1.37									

^a^The generation time (GT) or serial interval (SI) assumed in the reproduction number estimation

^b^Confidence interval

NR = Not reported

This table is also available as a .csv file as Additional file 5.

**Table 6:** **Reproduction numbers from novel influenza outbreaks**

Year(s)	Subtype	Study Population	Mean GT/SI^a^	Reproduction number (R)	95% CI^b^	Basic or Effective	Case definition	Reference	Year Published
1976	H1N1	New Jersey	1.9	1.20	1.1–1.4	Basic	Serologically confirmed illness	[122]	2007
2004–2006	H5N1	Vietnam	7	0.00	0–0.42	Effective	Laboratory confirmed illness	[119]	2008
2004–2006	H5N1	Indonesia	7	0.00	0–0	Effective	Laboratory confirmed illness	[119]	2008
2005	H5N1	Turkey	9.5	<1		Basic	Laboratory confirmed illness	[123]	2007
2005–2009	H5N1	Indonesia	6	0.1–0.25	0–0.4	Effective	Laboratory confirmed illness	[124]	2012
2006	H5N1	Indonesia	9.5	1.14	0.61–2.14	Basic	Laboratory confirmed illness	[123]	2007
Median reproduction number for novel influenza outbreaks: 0.34; Interquartile range 0.05–0.98									

^a^The generation time (GT) or serial interval (SI) assumed in the reproduction number estimation.

NR = Not reported.

^b^Confidence interval.

This table is also available as a .csv file as Additional file 6.

### 1918 influenza pandemic

The origins of the 1918 influenza A/H1N1 pandemic are unknown, and illnesses are thought to have occurred in three waves [1, 37]. The first wave began in the Northern Hemisphere in the spring 1918 [1]. A second wave of more intense transmission occurred concurrently in North America, Europe, and Africa in fall 1918, and a third and final wave occurred in some areas of the world during winter 1919 [37, 125]. The 1918 pandemic was the most deadly pandemic ever recorded, and an estimated 675,000 deaths occurred in the United States during the pandemic period. In contrast to seasonal influenza, which disproportionately affects the very young and old, those aged 20–40 years were especially affected [37].

**Figure 2:**
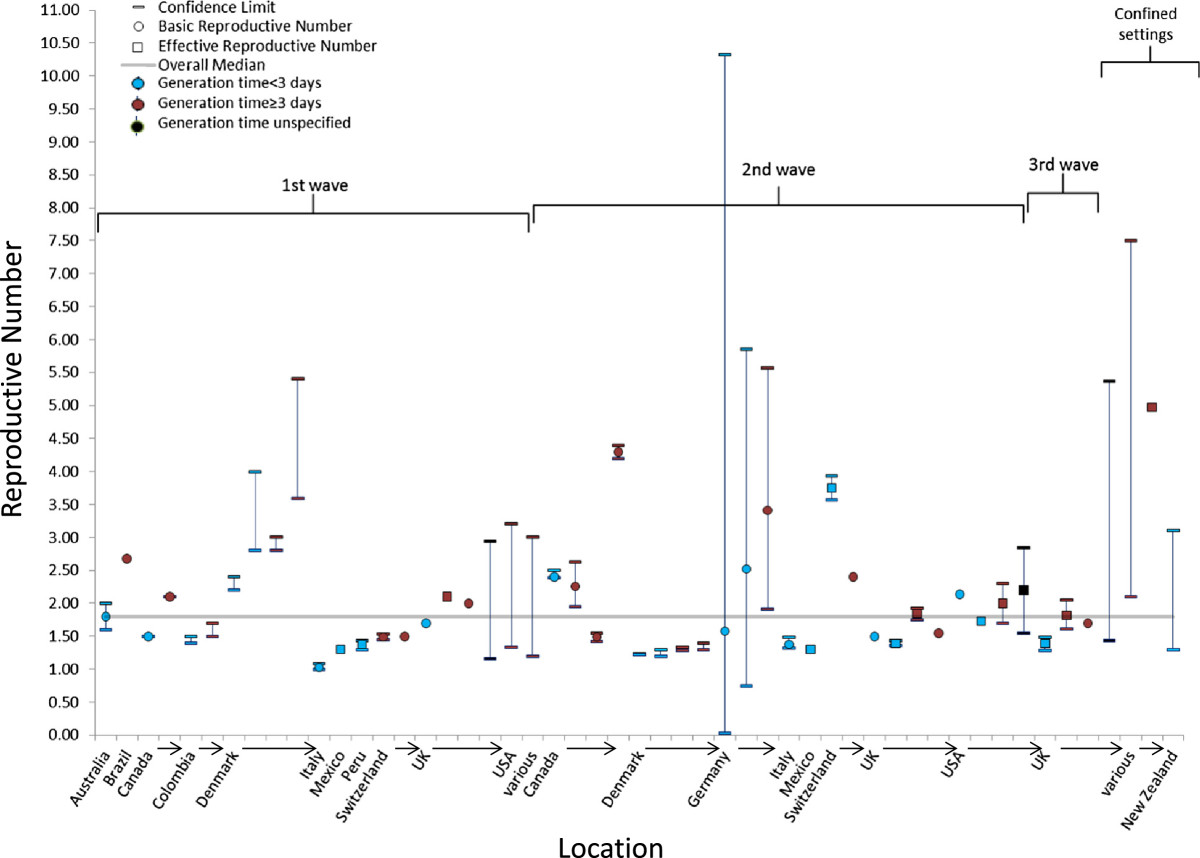
**Estimates of the reproduction number for the 1918 influenza A/H1N1 pandemic according to location, wave of illness, setting, and the serial interval or generation time assumed in the estimation method.** For individual studies, the single estimate or median of multiple estimates is shown as a circle for basic reproduction numbers or a square for effective reproduction numbers, and the range or confidence interval is denoted by brackets. Estimates of the reproduction number are color coded based on the generation time or serial interval used in calculations: red (<3 days), blue (≥3 days), or black (not reported or not used).

Twenty-four studies reported 51 separate 1918 pandemic values of R (Table 1; Figure 2). The median point estimate of R in the community setting for all waves of illness was 1.80 (IQR: 1.47–2.27) (Table 1). A higher median R value (R = 3.82; IQR: 2.68–4.84) was reported in confined settings, such as ships, military camps, and schools. The median values of R were similar between the first and subsequent waves of illness: the median value of R was 1.81 (IQR: 1.50–2.28) for the 1^st^ wave, 1.73 (IQR: 1.39–2.33) for the second wave, and 1.70 (IQR: 1.55–1.76) for the third wave (Table 1).

The majority of 1918 pandemic values for R were calculated for populations in Europe, which accounted for 58% of the R estimates included in this analysis. The mean generation time or serial interval used in the calculations to estimate R had a median value of 3.3 days, and the mean ranged from 1.5–6 days. Because the influenza virus was not discovered until 1931[1], all studies included in this review relied on reports of unconfirmed illness to identify those ill. A majority (65%) used pneumonia-and-influenza-related hospitalizations and deaths as the case ascertainment source (Table 1).

### 1957 influenza pandemic

The 1957 influenza A/H2N2 pandemic began in February 1957 in southern China and spread to Singapore and Hong Kong in April [1]. The virus was first isolated in the United States in June 1957 and was associated with a first wave [1, 41]. The peak of the pandemic occurred during the second wave in the Northern Hemisphere in October 1957 and was followed by a third wave in January 1958. An estimated 115,000 deaths occurred in the United States during the pandemic period [37].

**Figure 3:**
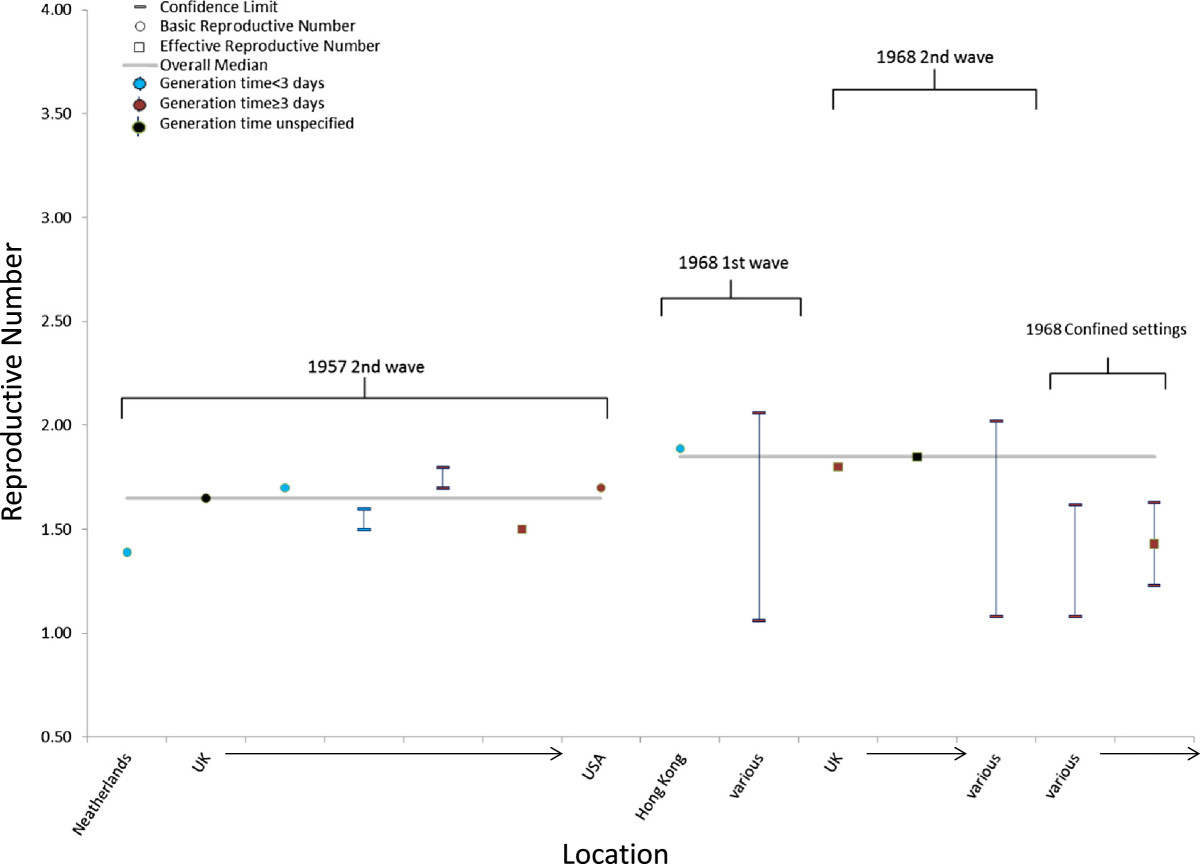
**Estimates of the reproduction number for the 1957 influenza A/H2N2 and the 1968 influenza A/H3N2 pandemics according to location, wave of illness, setting, and the serial interval or generation time assumed in the estimation method.** For individual studies, the single estimate or median of multiple estimates is shown as a circle for basic reproduction numbers or a square for effective reproduction numbers, and the range or confidence interval is denoted by brackets. Estimates of the reproduction number are color coded based on the generation time or serial interval used in calculations: red (<3 days), blue (≥3 days), or black (not reported or not used).

Six studies reported seven separate 1957 pandemic values of R (Table 2; Figure 3). The median point estimate of R in the community setting for the second wave of illnesses was 1.65 (IQR: 1.53–1.70). No R values were reported for confined settings or for the 1^st^ or 3^rd^ waves of illness.

A majority (86%) of 1957 pandemic R values were calculated for populations in Europe. The mean generation time or serial interval used in the calculations to determine R had a median value of 3.5 days, and the mean ranged from 2.6–4.1 days. All studies but one included in this review relied on an unconfirmed illnesses to identify those ill. The other study relied on the final attack rate as determined by serological methods (Table 2).

### 1968 influenza pandemic

The 1968 influenza A/H3N2 pandemic began in Hong Kong in July 1968. Large single waves were reported in the Northern Hemisphere between September 1968 and April 1969 (with peaks occurring in December and January) and in the Southern Hemisphere between June and September 1969. Some countries in the Northern Hemisphere, such as the United Kingdom, did not have an outbreak of H3N2 until the winter of 1969–70. In all, an estimated 110,000 deaths occurred in the United States during the pandemic period [37].

Four studies reported seven separate 1968 pandemic values of R (Table 3; Figure 3). The median point estimate of R in the community setting for all waves of illness was 1.80 (IQR: 1.56–1.85) (Table 3). Only two values for R in confined settings were reported, and the median value was 1.39. Two values of R were reported in a community setting during the first wave and three during the second wave. The median value of R during the 1^st^ wave was 1.56 and 1.68 during the 2^nd^ wave (Table 3).

The 1968 pandemic values for R were calculated among populations in diverse geographic locations, mainly because of one study that calculated separate values for over 25 locations, such as Africa, Asia, and South America (the overall estimate for R is included in Table 3) [43]. The mean generation time or serial interval used in the calculations to determine R had a median value of 4 days with little variation. The studies for the 1968 pandemic included in this review relied on a mix of laboratory-confirmed, unconfirmed illnesses, or serologically-confirmed infections to identify those ill (Table 3).

### The 2009 influenza pandemic

The 2009 influenza A/H1N1 pandemic began in Mexico in the late winter or early spring 2009 [44]. The United States and the United Kingdom experienced a first wave of illnesses in the spring followed by a second wave during the fall [4]. However, a number of other countries, especially in the Southern Hemisphere, only experienced a single wave of illnesses [100]. In all, an estimated 12,000 deaths occurred in the United States during the first year of pandemic circulation [126].

**Figure 4:**
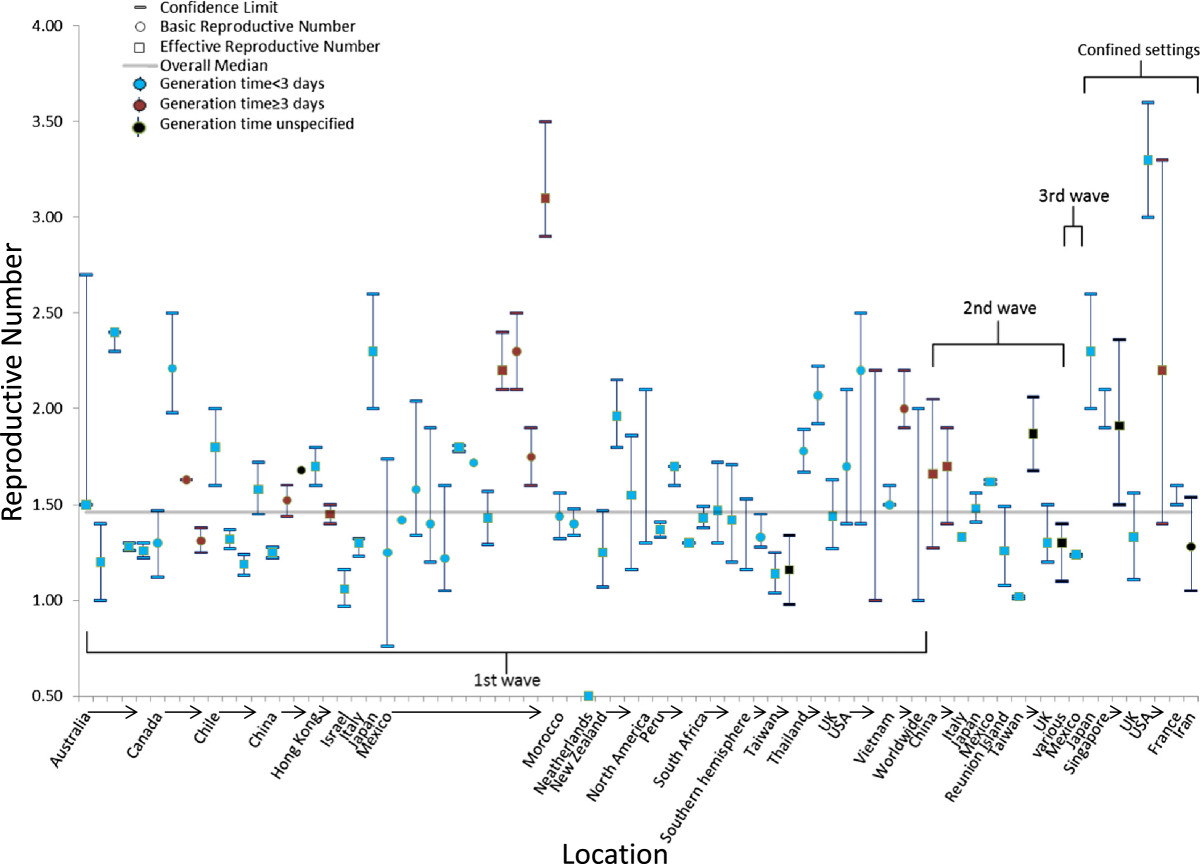
**Estimates of the reproduction number for the 2009 Influenza A/H1N1 pandemic according to location, wave of illness, setting, and the serial interval or generation time assumed in the estimation method.** For individual studies, the single estimate or median of multiple estimates is shown as a circle for basic reproduction numbers or a square for effective reproduction numbers, and the range or confidence interval is denoted by brackets. Estimates of the reproduction number are color coded based on the generation time or serial interval used in calculations: red (<3 days), blue (≥3 days), or black (not reported or not used).

Fifty-seven studies reported 78 separate 2009 pandemic values of R (Table 4; Figure 4). The median point estimate of R in the community setting for all waves of illness was 1.46 (IQR: 1.30–1.70) while a higher median R value (R = 1.96; IQR: 1.50–2.23) was reported in confined settings, such as military or summer camps, schools, and night clubs. The value of R was similar across the two distinct waves of illness: the median value of R was 1.47 (IQR: 1.31–1.71) for the first wave and 1.48 (IQR: 1.30–1.66) for the second wave (Table 4).

A majority of 2009 pandemic values for R were calculated for populations in North America (30%) and Asia (26%). The mean generation time or serial interval used in the calculations to determine R had a median value of 2.8 days, and the mean ranged from 1.9–7 days (Table 4). A majority of the studies included for the 2009 pandemic relied on either laboratory-confirmed illnesses (71%) or unconfirmed illnesses (24%) to identify those ill (Table 4).

### Seasonal influenza

Seasonal influenza causes sustained epidemics in the non-tropical areas of the Northern Hemisphere and Southern Hemisphere during their respective late fall to early spring months. Epidemics in the tropical regions occur sporadically but can be associated with the rainy season [1]. The mortality burden from influenza varies by season, and from 1976–2007, estimates of annual influenza-associated deaths in the United States from respiratory and circulatory causes ranged from 3,000 to 49,000 [3].

**Figure 5:**
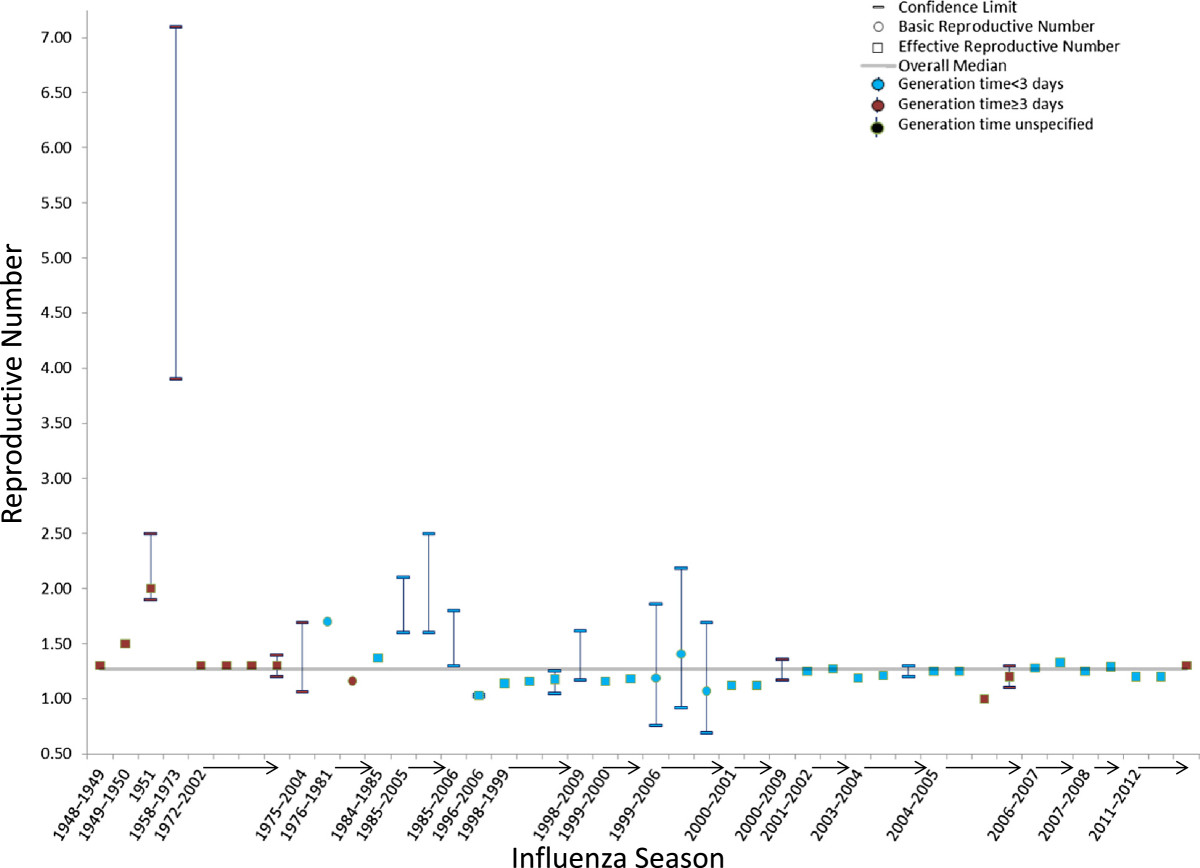
**Estimates of the reproduction number in the community for seasonal influenza epidemics according to location, wave of illness, and the serial interval or generation time assumed in the estimation method.** For individual studies, the single estimate or median of multiple estimates is shown as a circle for basic reproduction numbers or a square for effective reproduction numbers, and the range or confidence interval is denoted by brackets. Estimates of the reproduction number are color coded based on the generation time or serial interval used in calculations: red (<3 days), blue (≥3 days), or black (not reported or not used).

Twenty-four studies reported 47 separate seasonal epidemic values of R (Table 5; Figure 5). The median point estimate of R in the community setting for seasonal influenza was 1.27 (IQR: 1.19–1.37) while a higher median R value (R = 16.9) was reported in a British boarding school during the 1977–78 influenza season (Table 5). R values for seasons where H3N2 (R = 1.25; IQR: 1.18–1.27) or H1N1 (R = 1.25; IQR: 1.18–1.35) predominated were equivalent (Table 5).

A majority of seasonal influenza values for R were calculated for populations in Israel (35%), Europe (25%), and North America (21%). The mean generation time or serial interval used in the calculations to determine R had a median value of 3.0 days, and the mean ranged from 2.0–7.0 days (Table 5). A majority of the studies included for seasonal influenza relied on unconfirmed illnesses or deaths (79%); the reminder relied on either laboratory-confirmed illnesses or hospitalizations or serologically-confirmed infections (Table 5).

### Human infections with non-human influenza viruses

Human infections with novel or non-human influenza viruses (also known as zoonotic influenza viruses) are rare but can result in a pandemic if sustained person-to-person transmission occurs and the population has little or no pre-existing population immunity to the virus. Therefore, instances of infection with non-human influenza viruses are investigated thoroughly to assess the transmissibility of the virus. The largest number of novel influenza cases at the time of this review was from the ongoing influenza A/H5N1 outbreak centered in Southeast Asia and the Middle East. From January, 1, 2003 to February 15, 2013, 620 laboratory-confirmed cases have been reported to the WHO, of which 367 have died [127]. Another large outbreak of novel influenza occurred in 1976 in Fort Dix, New Jersey, which was caused by an influenza A/H1N1 virus similar to those found circulating in swine [122].

Four studies estimated the values of R for the A/H5N1 and A/H1N1 outbreaks (Table 6). Four out of six estimates (67%) of R were less than one, and the highest R estimate (R = 1.2) was for the 1976 A/H1N1 outbreak in a New Jersey military camp (a confined setting) (Table 6).

A majority of novel A virus R values were calculated for populations in Southeast Asia (67%), indicative of where the bulk of A/H5N1 bird-to-human transmission occurs. The mean generation time or serial interval used in the calculations to determine R had a median value of 7.0 days, and the mean ranged from 1.9–9.5 days (Table 6). All studies relied on either laboratory-confirmed illness or serological-confirmed infection (Table 6).

## Discussion

In this review, the median R values reported for the four pandemics and seasonal influenza varied between 1.27–1.8 while R values for novel influenza were generally below 1. We found the highest median reproduction number associated with the 1918 and the 1968 influenza pandemics (both 1.8), followed by the 1957 pandemic (1.65), the 2009 pandemic (1.46), seasonal influenza epidemics (1.27), and novel influenza outbreaks. A majority of R values published were for either the 1918 pandemic or the 2009 pandemic; the 1957 and 1968 pandemics had the fewest published studies. Researchers calculated values for R for a variety of locations and utilized many different case definitions, ascertainment methods, and assumptions about the generation time or serial interval.

The approximate basic reproductive numbers for some common infectious diseases range from 12–18 for measles, 12–17 for pertussis, and 4–7 for mumps, polio, rubella, and smallpox [12]. These values are much higher than what has been reported for influenza, and most R values reported in this review ranged from 1.0–2.0. However, the overall clinical attack rate and peak daily incidence of an outbreak, which measures the potential burden on healthcare services and school and workplace absenteeism, are very sensitive to changes in the value of R within this range. Past research utilizing a number of assumptions on the symptomatic ratio, contact patterns, and seeding has estimated that the cumulative clinical attack rates for a pandemic when R = 1.3 ranged from 15%–21% and increased to 34%–42% for R = 2.0 [10, 11]. Similarly, the peak daily attack rate is 0.5% for R = 1.3 and 2.2% for R = 2.0 [10]. Therefore, with only an absolute difference in R of 0.7, the clinical attack rates in these studies more than doubled and the peak daily incidence more than quadrupled.

Differences in the value of R within this range also affect the evaluation of potential mitigation strategies (e.g., school closures, vaccination, household isolation) for influenza pandemics. Analysis of strategies to mitigate an influenza pandemic have found that the effectiveness of non-travel-related control policies, such as school closures, household quarantine, and vaccination, would decrease as the value of R increases from 1.0 to 2.0 [10]. The success of various vaccination strategies would also be more likely for values of R < 1.7 [10, 11]. Therefore, the small variations in pandemic R estimates found in this analysis can have important implications for the overall impact and success of mitigation efforts for an influenza pandemic. This finding highlights the importance of making precise estimates of R early in a pandemic. Further research should focus on refining methods that allow for early, robust estimates of R.

The results of this analysis reinforce the idea that R is a measure that captures the transmissibility of an influenza virus in the population under study and is not an intrinsic value. The inputs for its calculation can include the population contact rate, the probability of infection per contact, the duration of illness, and the percentage of the population that is susceptible which is affected by the characteristics of the population under study. Therefore, the variations in the value for R for the same pandemic or seasonal outbreak are expected and may be due to the underlying social and socio-demographic factors of the population studied, public health interventions, and geographical or climatic factors of the location. These variations include the percentage of the source’s population under 18 years old; differences in contact patterns between age groups, which vary by country [128, 129]; and differences in population susceptibility profiles, which varied by age group for the 2009 pandemic [130]. Another important factor that may contribute to the variation is the season from which data used to estimate R is collected. While the effect of weather on the transmissibility of influenza has not been fully explored, some studies have shown that the level of absolute humidity is inversely correlated with influenza transmissibility [131, 132]. Therefore, estimates of R should be interpreted in the context of the population under study and the season in which data was collected and direct comparisons of R between populations should be undertaken with caution.

Variations in the estimated values of R may also be driven by changes in surveillance intensity in the same country over time. If a country suddenly improves its surveillance system in response to a pandemic and is better able to identify cases, then the number of cases being reported will increase, even though the actual number of cases occurring will not have changed. This increase in the reported number of cases may increase the estimated R as the growth rate of the outbreak will increase [86]. Conversely, the value of R could be artificially lowered if countries implement changes in surveillance practices that result in a lower number of identified cases, such as reducing screening recommendations, or have their surveillance systems overwhelmed. This effect was seen in the United States during the 2009 pandemic, when influenza testing for every case became unfeasible and testing recommendations were changed [4].

One of the more important methodological assumptions that can have a large impact on the estimated value of R is the length of the serial interval or generation time used during the estimation of R. Longer serial intervals have previously been associated with higher estimates of R when compared to estimates from the same dataset using shorter serial intervals [9]. In this analysis, estimates of R from the 1918, 1957, and 1968 pandemics utilized higher serial interval values than were used for the 2009 pandemic or for seasonal influenza. Additionally, higher values of R from the 2009 pandemic often were estimated using a generation time or serial interval of 3 days or more (Figure 4). Therefore, the estimates of R included in this analysis should be interpreted in the context of the serial intervals or generation times used in the estimation method. Like R, the values for the generation time or the serial interval can vary by the source population. Therefore, researchers estimating the values of R should strive to use standard estimates of the serial interval or generation time for influenza or at least include common values in a sensitivity analysis. This will help with the comparability of R values across studies and may aid in the correct interpretation of R estimates. An additional way in which estimates of R may be biased up or down lies in the choice of estimation procedure itself. Chowell et al. showed that estimates of R obtained using simple epidemic mathematical models varied considerably as the model increased in complexity (e.g. the addition of a period of infection latency or an age-structured population) [35].

Although we found no difference in the value of R for studies using confirmed cases versus unconfirmed cases in the estimation method, the trade-off between the accuracy of the less specific but more efficient and cost effective syndromic data compared to laboratory-confirmed influenza infections is unknown. The incubation periods of non-influenza respiratory pathogens that co-circulate with influenza (e.g. respiratory syncytial virus or rhinovirus) range from a median of 1.9–5.6 days; estimates of R for influenza could either be overestimated or underestimated during periods of co-circulation, depending on the intensity and identity of the co-circulating respiratory pathogen [87]. Future research should focus on estimation of R using laboratory-confirmed cases and hospitalizations and should provide estimates from syndromic data for comparison.

Most studies included in this analysis focused on 1918 or the 2009 pandemic. Only a small number of estimates of the reproduction number have been reported for the two other pandemics of the 20^th^ century (1957 and 1968). As a consequence, there is still insufficient information to fully clarify the transmission dynamics of the 1957 and 1968 pandemics. Because historical data are available for these pandemics, future research should focus on estimations of R for the 1957 and 1968 pandemics to better understand the characteristics of these pandemics.

This study generally found higher reproduction numbers for confined settings, such as schools, military bases, or night clubs, except for estimates from the 1968 pandemic. Because confined settings increase the intensity of transmission by increasing contact rates among those ill and well, the values of R presented for outbreaks in confined settings are likely to be much higher than values of R estimated for the community and should be interpreted accordingly. While the estimation of R in confined settings may be useful for the assessment of the upper bounds of transmissibility, its value is not directly comparable to estimates of R made in the community setting.

This review found, with one exception, a high degree of consistency in the estimated values of R for seasonal influenza epidemics. The only notable exception was the extremely high R values estimated for an outbreak of influenza A (H1N1) in 1978 at a small British boarding school with 763 male students aged 10–18 who were mostly full boarders [133]. The results of this analysis suggest that the extreme R values reported for this outbreak are not typical of seasonal or pandemic influenza and instead may be the result of the lack of pre-existing immunity among the students to the strain of influenza A (H1N1) that caused the outbreak, the extremely high contact rates likely among a group of boarded students, or a study artifact related to the small number of students in the study population [13, 106, 133]. Additionally, the median R value of seasonal influenza (R = 1.27) is well below the median values seen during the four pandemics examined in this report. The consistency of seasonal R values is even more remarkable given the wide variety of estimation methods, data sources, and assumptions used in the studies included here. However, the majorities of seasonal influenza estimates were from a small number of countries. Estimates of R from countries in Africa, Asia, and South America are also needed to determine if values of R for seasonal influenza epidemics are affected by geographic and social factors.

This systematic review is subject to at least three limitations. First, we combined estimates for the basic and effective reproductive numbers when presenting the median estimates in this study. Even though these values measure transmission in populations with differing levels of underlying population immunity, some papers included in this review did not clearly differentiate between basic and effective reproductive numbers or state the required population immunity assumptions when reporting basic reproductive numbers. Therefore, we choose to present summary values for the basic and effective reproductive numbers together to simplify the results. The tables include whether the reproductive number estimate was reported as basic or effective for each study. Second, we did not assess included studies for the type or quality of their methodology or the risk of study bias. Finally, we only included published estimates of the reproductive number, which may not be representative of unpublished reproductive number values.

## Conclusions

In this review, we explored the ranges and uncertainty of the values of R estimated for seasonal, pandemic, and novel influenza. We found that values of R changed over the course of a pandemic but the effect of the waves varied. The value of R is not constant and may be affected by mitigation strategies, the season, and the population under study. The values of R found in this analysis represent the difference between a pandemic that is controllable with less intensive mitigation strategies and would cause moderate amounts of illness to a pandemic that would require very intensive mitigation strategies and would cause greater amounts of illness. Continued monitoring of R during outbreaks of human infections with non-human influenza viruses and in various settings throughout future pandemics will be required to fully understand the effects of mitigation, geography, and season.

## Electronic supplementary material

Additional file 1: Reproduction Numbers from the 1918 Influenza A/H1N1 Pandemic.(CSV 6 KB)

Additional file 2: Reproduction Numbers from the 1957 Influenza A/H2N2 Pandemic.(CSV 2 KB)

Additional file 3: Reproduction Numbers from the 1968 Influenza A/H3N2 Pandemic.(CSV 2 KB)

Additional file 4: Reproduction Numbers from the 2009 Influenza A/H1N1 Pandemic.(CSV 10 KB)

Additional file 5: Reproduction Numbers from Seasonal Influenza Epidemics.(CSV 5 KB)

Additional file 6: Reproduction Numbers from Novel Influenza Outbreaks.(CSV 1 KB)

Below are the links to the authors’ original submitted files for images.

Authors’ original file for figure 1

Authors’ original file for figure 2

Authors’ original file for figure 3

Authors’ original file for figure 4

Authors’ original file for figure 5
