# Supplementary figures and images for: Estimates of the reproduction number for seasonal, pandemic, and zoonotic influenza: a systematic review of the literature
Source: BMC Infect Dis. 2014 Sep 4;14:480. doi: 10.1186/1471-2334-14-480 (PMC4169819; doi:10.1186/1471-2334-14-480)

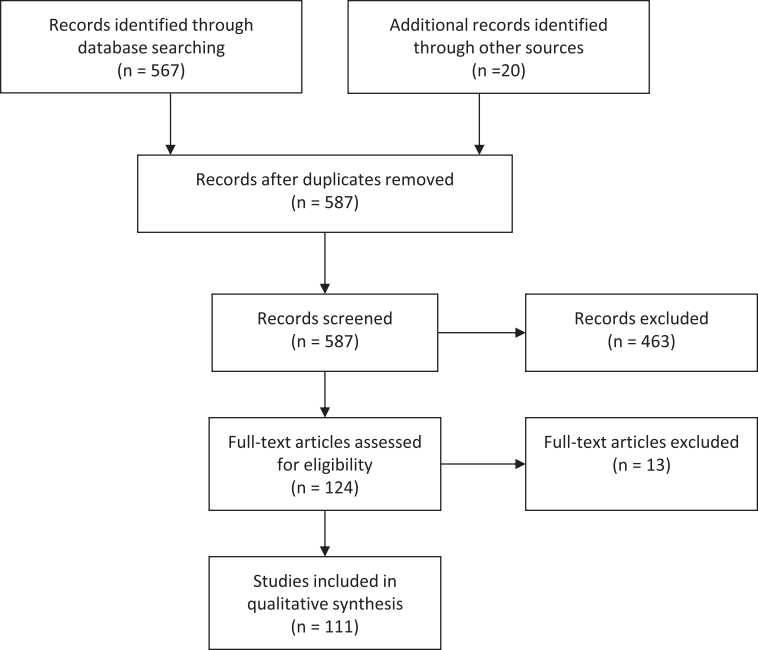

Supplement: Supplementary file 7 — Authors’ original file for figure 1 [file 12879_2014_3799_MOESM7_ESM.pdf]

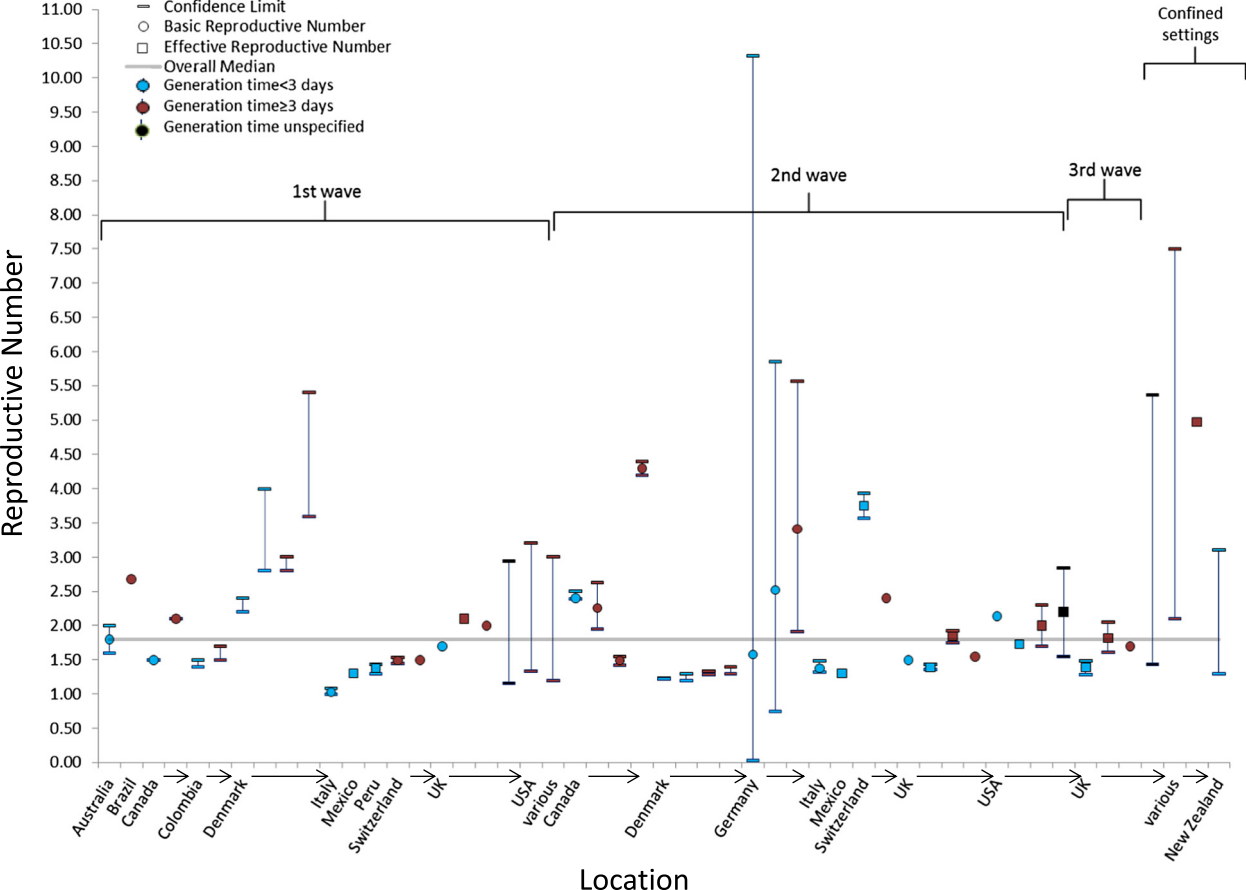

Supplement: Supplementary file 8 — Authors’ original file for figure 2 [file 12879_2014_3799_MOESM8_ESM.pdf]

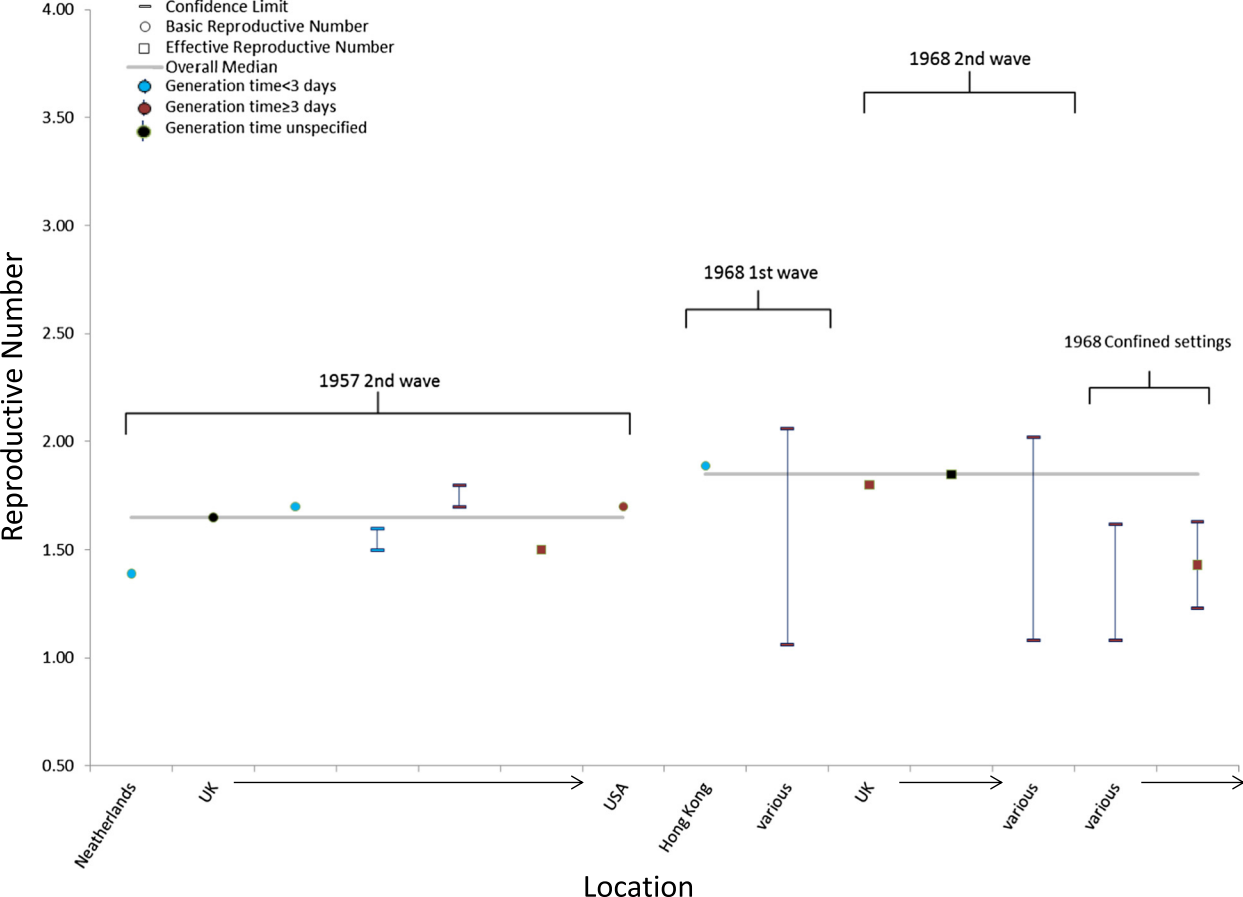

Supplement: Supplementary file 9 — Authors’ original file for figure 3 [file 12879_2014_3799_MOESM9_ESM.pdf]

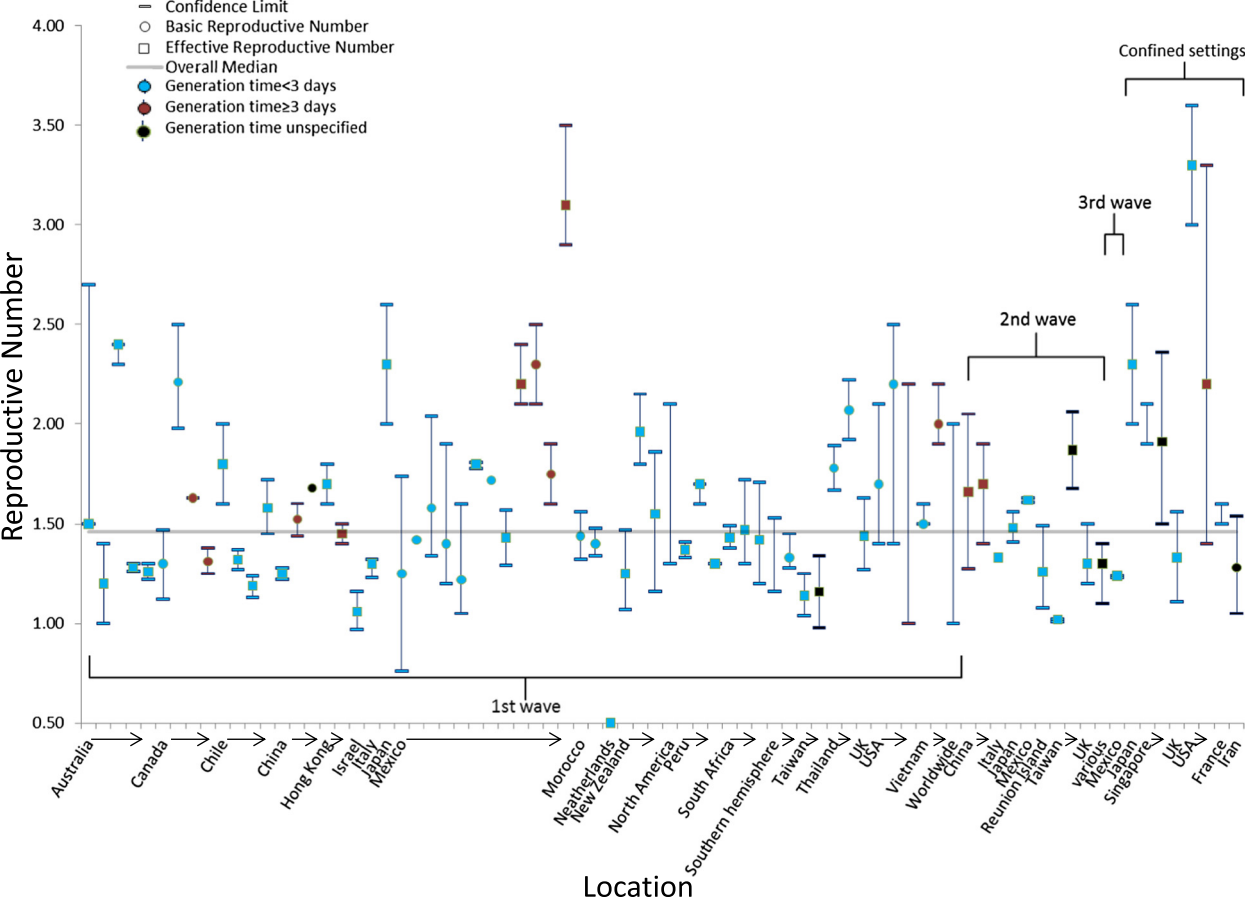

Supplement: Supplementary file 10 — Authors’ original file for figure 4 [file 12879_2014_3799_MOESM10_ESM.pdf]

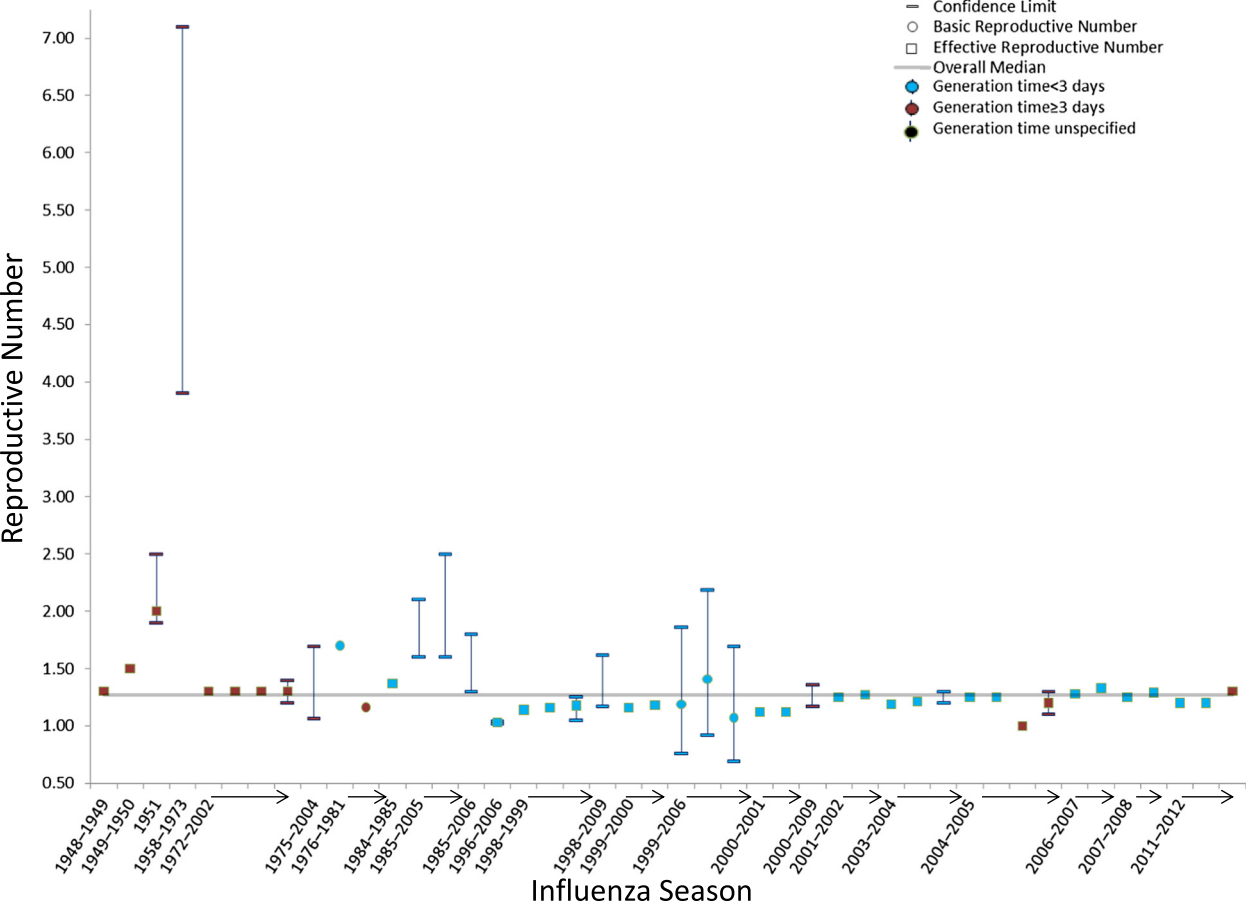

Supplement: Supplementary file 11 — Authors’ original file for figure 5 [file 12879_2014_3799_MOESM11_ESM.pdf]
